# Supplementary material for: Vaginal microbiota and personal risk factors associated with HPV status conversion—A new approach to reduce the risk of cervical cancer?
Source: PLoS One. 2022 Aug 9;17(8):e0270521. doi: 10.1371/journal.pone.0270521 (PMC9362946; doi:10.1371/journal.pone.0270521)
Supplement: S1 Text — (DOCX) [file pone.0270521.s004.docx]

**SUPPLEMENTARY MATERIAL**

**METHODS**

**Vaginal microbiome pilot program recruitment process**

**I. Recruitment background**: The purpose of the recruitment is to collect vaginal microbiome samples composition to identify robust biomarkers that could be used to prevent persistent HPV infections or to promote HPV clearance.

**II. Recruitment period**: May 25, 2020, May 31^st^, 2021,

(The following points are related to Activity 1 and Activity 2)

**III. Recruitment channel**: Online

**IV. Participant eligibility**: non-pregnant, non-lactating women who have had sex at least once in their lifetime.

**V. Advertisement channels**: BGI’s eHealth platforms, such as CanSeq live broadcast and CanSeq WeChat public account.

**VI. User coverage**: users of the WeChat instant messaging

Activity 1: Create a “vaginal microbiome pilot program” WeChat instant-messaging group
Activity 2: Create the WeChat interface that allows eligible participants to place an order for the HPV self-sampling collection kits used for this pilot program.

**VII. Specific Events**

**7.1 Participation requirements**

1. Interested subjects fill in an application questionnaire (Details in Annex I)

2. The pilot program project managers review the application questionnaires and contact eligible subjects to confirm their recruitment

3. Confirmed participants are pulled into a WeChat instant-messaging group where they have access to a link that prompts them to place the order for the self-sampling collection kits (participants give a deposit of 199 CNY which is fully refunded after the samples are sent for testing within a week of reception)

4. Participants receive the self-sampling collection kits required for the test in their designated address, carry out the cervicovaginal mucus self-sampling and collection, and post the sample back to the central testing laboratory.

5. After 30 days a test report will be issued, participants can access the information by using the CanSeq eHealth mini-program in WeChat’s instant-messaging platform.

**7.2 Participation Benefits**

1. Participants obtain their reproductive tract microecological test results (with a commercial value of 1499 CNY) along with interpretation support.

2. Participants obtain one-to-one gynecological consultation from registered medical professionals;

**VIII. Specific items of activity II**

**8.1 Participate in the program**

1. Participants are required to attend the recorded lectures (webinars) related to reproductive tract microecology which can be accessed through the CanSeq eHealth mini-program

2. Participants are required to enter the WeChat group of the vaginal microecology pilot program

3. Participants are required to place an order for the sample collection devices using the WeChat interface

4. Participants are required to carry out a cervicovaginal mucus self-sampling using the collection kit

5. Participants are required to return the samples to the central testing laboratory via the prepaid postal office.

5. After 30 days a test report will be issued, participants can access the information by using the CanSeq eHealth mini-program in WeChat’s instant-messaging platform.

**8.2 Activity Benefits**

1. Participants obtain their HPV genotyping test results (with a commercial value of 499 CNY) along with interpretation support.

2. Participants gain access to medical-lecture webinars on reproductive tract health

**X. Vaginal microbiome testing**

10.1 Test content

1. Analysis of the vaginal microbiome test results, included a classification of the community state types (CST) I, II, III, IV, and V of the vaginal microbiome, and a description and interpretation of different results;

2. Analysis of the vaginal micro-ecology flora composition and localization;

3. Identification of reproductive tract harmful microorganisms in the vaginal microbiome;

10.2 Test reporting was shown in supplementary 3 Metagenomics﻿.

XI. Summary of Frequently Asked Questions (see Annex II)

**Annex I. Reproductive tract microecology test application form**

Introduction: The dysregulation of the reproductive tract microenvironment increases the probability of persistent HPV infection, which is closely related to cervical disease and even the development of cervical cancer. Would you like to check the microecological health of your reproductive tract? Come and join the participation group! We will send you a free test kit!

1. Name:

2. Gender:

3. Mobile phone number:

4. WeChat ID:

5. Have you had or do you currently have an HPV infection? (Please specify the serotype):

6. How long have you been infected with HPV?

7. How long have you been on the eHealth platform?

8. Have you updated your identificiation card?

9. What services would you like to be provided on the eHealth platform?

**Annex II. Frequently Asked Questions**

1. When will the participation results come out?

Answer: After completing the application questionnaire and uploading the diagnostic report, BGI staff will review the information and will inform the participants about their application status via WeChat and SMS notification.

2. What do you need to do after successful registration?

Answer: You will be invited to join the activity group of “BU Golden Belt” through a WeChat mini-program. BGI staff will guide you to arrange for the delivery of products (HPV testing & reproductive tract microecological testing products).

Please read the product manual, and analyze the kit. In case of questions, please contact the administrators of the program. Collect the cervicovaginal mucus samples as per the indications and send the tests back to BGI’s laboratory using the pre-paid postal service.

3. When will the test results come out?

Answer: HPV test results will be available in 5 working days, and the reproductive tract microecology test in 30 natural days.

4. After I receive my test results, how can I schedule an appointment with the doctor?

Answer: For discussions regarding the HPV test please use the CanSeq mini-program to communicate with a medical professional in the “doctor consultation” section and for the vaginal microecology results, reach out to the group @doctor via WeChat.

**Eligible participants**

1. Women who have had sex at least once in their lifetime.

2. Non-pregnant

3. Non-lactating

**Sampling considerations**

1. Participants can collect a sample before or after taking a bath or shower – this does not affect the results of the test

2. Participants should avoid sex for 24 hours before sampling

3. Participants should not use vaginal douches or use vaginal medicines for at least 48 hours before sampling

4. Participants experiencing their menstrual period, should wait for 3 days after the end of their menstrual period before taking a sample

**Delivery considerations**

1. What do I do if the swab is too long to put inside the test tube?

Answer: After self-sampling, the swab should be broken according to the operating instructions.

2. How do pack the samples?

Answer: The test tube goes in the buffer bag, and the buffer bag goes in the transport bag. The transportation bag should be sealed.

3. What do I do with the QR code stickers?

Answer: Please use a single sticker on the external transportation bag. The second sticker is spare.

4. Should I return the product instructive along with the sample?

Answer: No

5. How should I send the samples?

Answer: Please schedule the package pickup via SUN EXPRESS, with the “pay on delivery” option.

6.What is the delivery address? How do I keep track of the delivery?

Answer: The instructions are available on the "Order Details" of Golden Ribbon Official Account "Mall"
