# Supplementary material for: Vaginal microbiota and personal risk factors associated with HPV status conversion—A new approach to reduce the risk of cervical cancer?
Source: PLoS One. 2022 Aug 9;17(8):e0270521. doi: 10.1371/journal.pone.0270521 (PMC9362946; doi:10.1371/journal.pone.0270521)
Supplement: S2 Text — (DOCX) [file pone.0270521.s005.docx]

**Personal Reported Clinical Factors**

1. Personal Information (7 questions)

1.1. Year of birth: _____

1.2. Height: _______cm

1.3. Weight: _______kg

1.4. Education level: infection rate is related

- Doctorate degree (or above)
- Master's degree
- Bachelor's degree
- College degree
- Technical school (completed)
- Technical school (incomplete)
- High-school
- Some high-school (did not graduate)
- Elementary school (completed)
- No formal education or some elementary school (incomplete)

1.5. Occupation:

- Student
- Unemployed: homemaker, or retired
- Worker
- Farmers
- Workers (directly involved in production)
- Regular workers in the business, service, or entertainment industries
- Self-employed workers, small traders, small business owners, sellers
- Office staff, white-collar workers, police officers
- Scientific and technical personnel, teachers, researchers, artists, or people in the media and advertising industry.
- Medium to large business owners, managers, factory directors, bosses (including shop owners through digital platforms)
- Public servants, government officials
- Others, please specify——

1.6, Monthly net income: __________ (RMB)

- Less than 1000 yuan
- 1000 to 3000 yuan
- 3000 to 5000 yuan
- 5000 to 10000 yuan
- 10,000 to 30,000 yuan
- 30,000 to 100,000 yuan
- More than 100,000 yuan

1.7. Location [use mini program to pick the right location]

**2. Personal current and history of the disease (7 factors)**

2.1. Are you anemic (with lower than 100 g/L hemoglobin levels)?

- Yes
- No

2.2. Have you suffered from any reproductive tract infection, such as bacterial vaginitis, candida vaginitis, trichomonas vaginitis, condyloma acuminatum, syphilis, gonorrhea, genital herpes, or others in your lifetime?

- Yes
- No

2.3. Are you diagnosed with endocrine diseases, such as diabetes and/or thyroid disease?

- Yes
- No

2.4. Are you currently suffering from any metabolic diseases, such as hyperglycemia, hyperlipidemia, and/or hyperuricemia?

- Yes
- No

2.5. Do you have a history of cancer (head, neck, vagina, vulva, or anus)?

- Yes
- No

2.6. Do you have any immediate family members (such as parents, children, and/or siblings) with a history of cancer?

- Yes
- Mo

2.7. Do you have a diagnosed mental illness (such as bipolar disorder, mania, depression)?

- Yes
- No

**3. Nutrition behavior assessment (The reference intake can be consulted in the attachment from the Chinese dietary balance index DBI_16]** (Take into account your diet over the past week and choose an average daily intake).

3.1. Cereals, tubers and beans (complex carbohydrates) consumption:

| Intake | Score |
| --- | --- |
| ＜35g | -12 |
| 35-50g | -11 |
| 50-65g | -10 |
| 65-80g | -9 |
| 80-95g | -8 |
| 95-110g | -7 |
| 110-125g | -6 |
| 125-140g | -5 |
| 140-155g | -4 |
| 155-170g | -3 |
| 170-185g | -2 |
| 185-200g | -1 |
| 200-250g | 0 |
| 250-265g | 1 |
| 265-280g | 2 |
| 280-295g | 3 |
| 295-310g | 4 |
| 310-325g | 5 |
| 325-340g | 6 |
| 340-355g | 7 |
| 355-370g | 8 |
| 370-385g | 9 |
| 385-400g | 10 |
| 400-415g | 11 |
| ＞415g | 12 |

[Cereals, tubers, and beans]


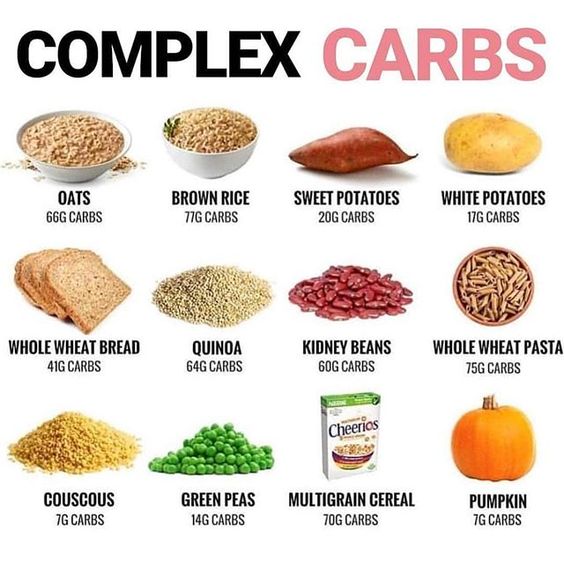


3.2. Vegetables and fruits:

(1) Vegetables

| Intake | Score |
| --- | --- |
| ≥400g | 0 |
| 320-399g | -1 |
| 240-319g | -2 |
| 160-239g | -3 |
| 80-159g | -4 |
| 1-79g | -5 |
| 0g | -6 |


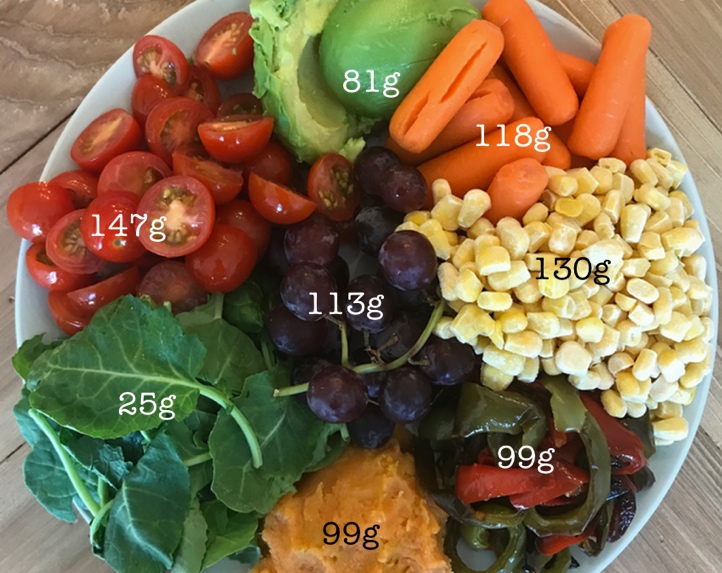


(2) Fruits

| Intake | Score |
| --- | --- |
| ≥200g | 0 |
| 160-199g | -1 |
| 120-159g | -2 |
| 80-119g | -3 |
| 40-79g | -4 |
| 1-39g | -5 |
| 0g | -6 |


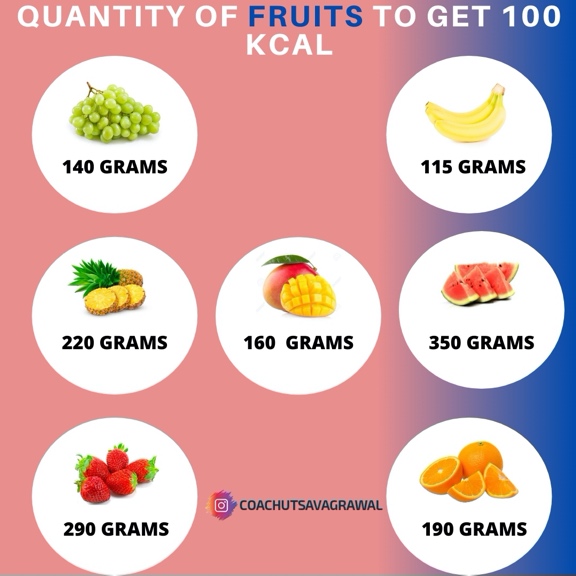


3.3. Milk and dairy products and soy milk and soy products

(1) Milk and dairy products

| Intake | Score |
| --- | --- |
| ≥300g | 0 |
| 240-299g | -1 |
| 180-239g | -2 |
| 120-179g | -3 |
| 60-119g | -4 |
| 1-59g | -5 |
| 0g | -6 |


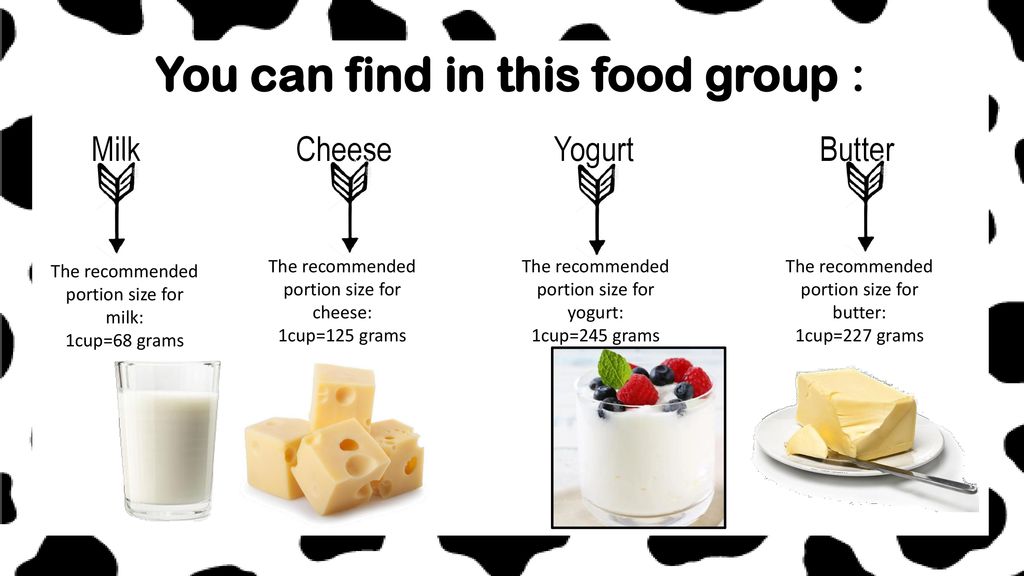


(2) Soybean milk and soy products

| Intake | Score |
| --- | --- |
| ≥15g | 0 |
| 12-14g | -1 |
| 9-11g | -2 |
| 6-8g | -3 |
| 3-5g | -4 |
| 1-2g | -5 |
| 0g | -6 |


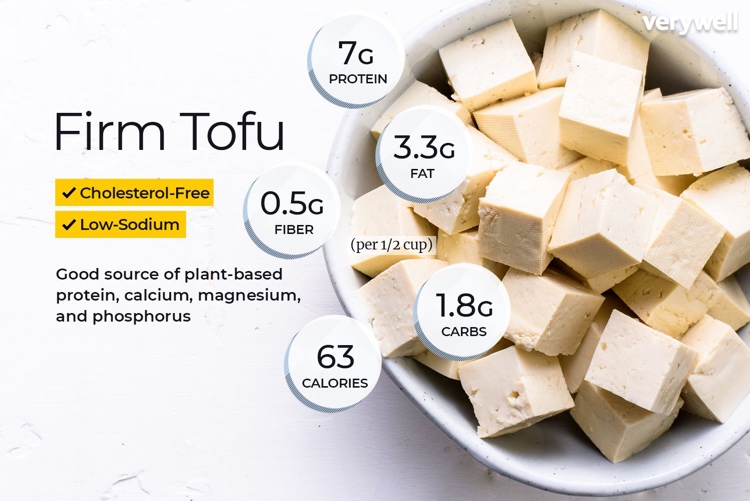


3.4. Animal food

(1) Red meat and poultry

| Intake | Score |
| --- | --- |
| 0g | -4 |
| 1-15g | -3 |
| 16-30g | -2 |
| 31-45g | -1 |
| 46-55g | 0 |
| 56-70g | 1 |
| 71-85g | 2 |
| 85-100g | 3 |
| ＞100g | 4 |


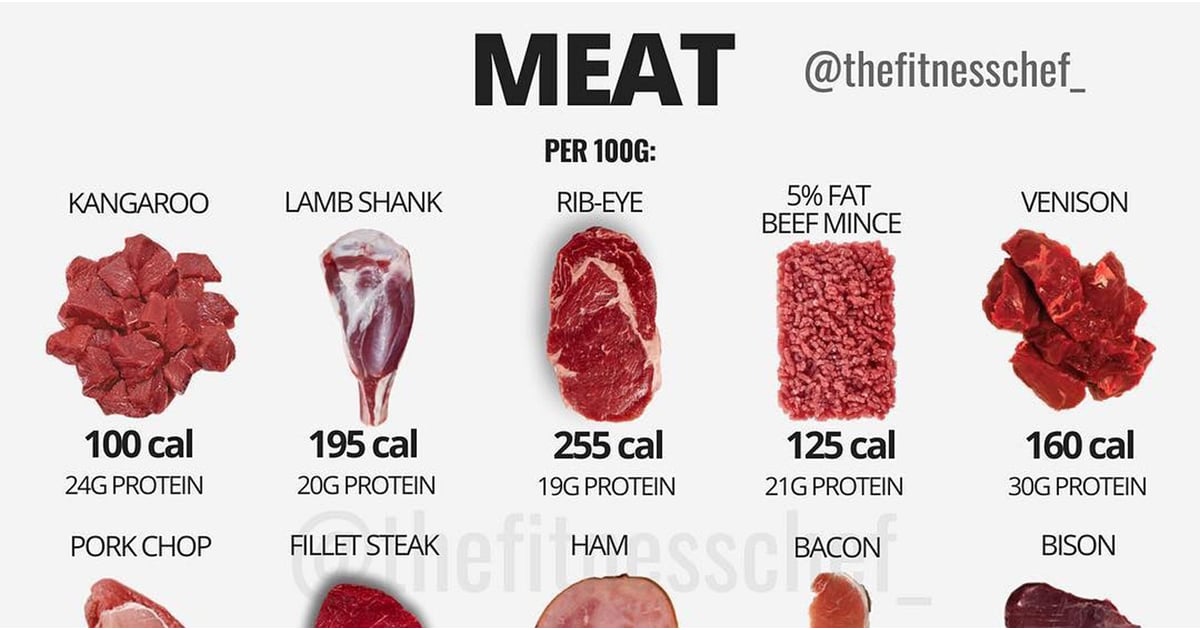


(2) Fish, shrimp, and other kinds of seafood

| Intake | Score |
| --- | --- |
| 5g | -4 |
| 5-19g | -3 |
| 20-34g | -2 |
| 35-49g | -1 |
| ≥50g | 0 |


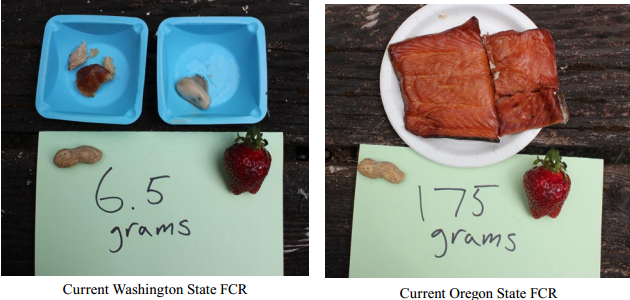


(3) Eggs

| Intake | Score |
| --- | --- |
| 0g | -4 |
| 1-10g | -3 |
| 11-20g | -2 |
| 21-30g | -1 |
| 31-50g | 0 |
| 51-60g | 1 |
| 61-70g | 2 |
| 71-80g | 3 |
| ＞80g | 4 |


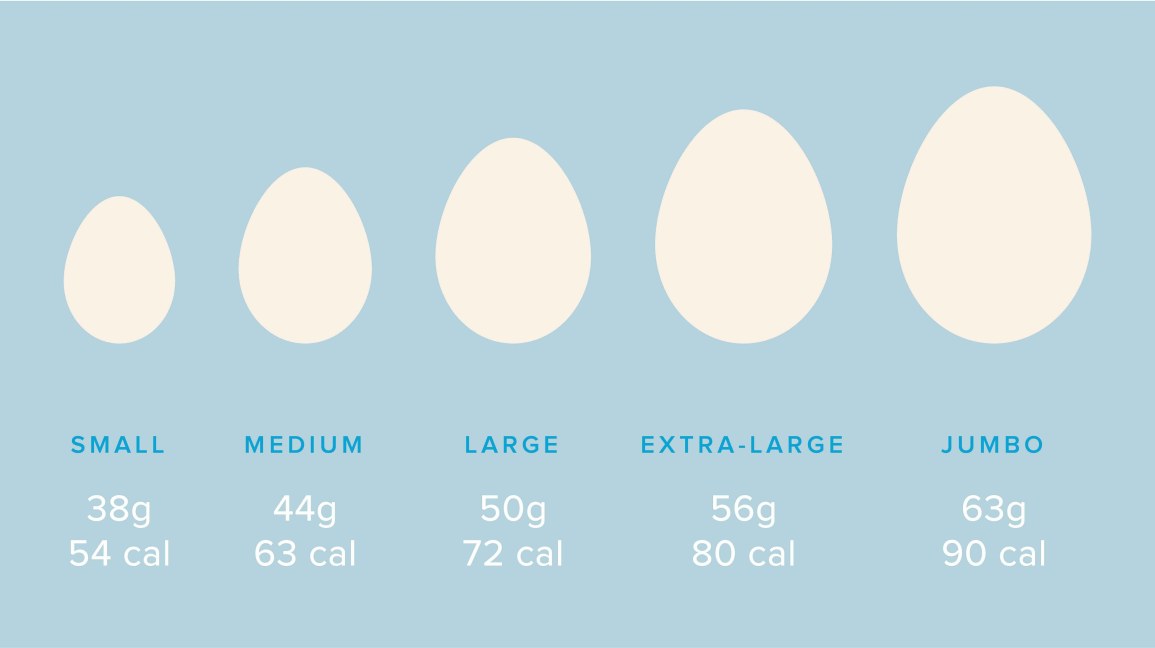


3.5. Energy-free foods

(1) Edible oil

| Intake | Score |
| --- | --- |
| ≤25g | 0 |
| 26-30g | 1 |
| 31-35 | 2 |
| 36-40 | 3 |
| 41-45 | 4 |
| 46-50 | 5 |
| ＞50g | 6 |
| I don't know |  |

If you chose "I don't know", please select a word that describes your self-perceived oil consumption

- heavy
- average
- little


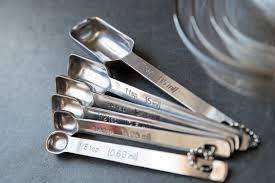


(2) Alcoholic beverages

15g of alcohol = 450 ml of beer or 150 ml of wine

30g of alcohol = XXX mL of <38°liquor

50g of alcohol = XXX ml of 38°liquor

| Intake | Score |
| --- | --- |
| ≤15g | 0 |
| 16-25g | 1 |
| 26-35g | 2 |
| 36-45g | 3 |
| 46-55g | 4 |
| 56-65g | 5 |
| ＞65g | 6 |
| I don't know |  |

If you chose "I don't know", please select indicate how often do you have an alcoholic drink?

- Frequently (over 2 times per week)
- Occasionally (one time per week or less)
- Never


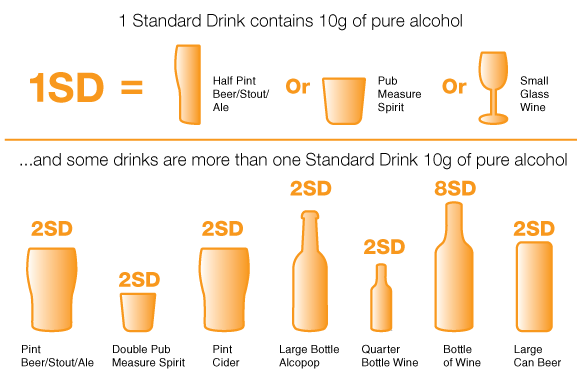


3.6. Condiments

(1) Sugar

| Intake | Score |
| --- | --- |
| ≤25g | 0 |
| 26-30g | 1 |
| 31-35g | 2 |
| 36-40g | 3 |
| 41-45g | 4 |
| 46-50g | 5 |
| ＞50g | 6 |
| I don't know |  |

If you chose "I don't know", please answer how often do you eat sweets or drink sugary drinks?

- Often
- Moderately
- Rarely


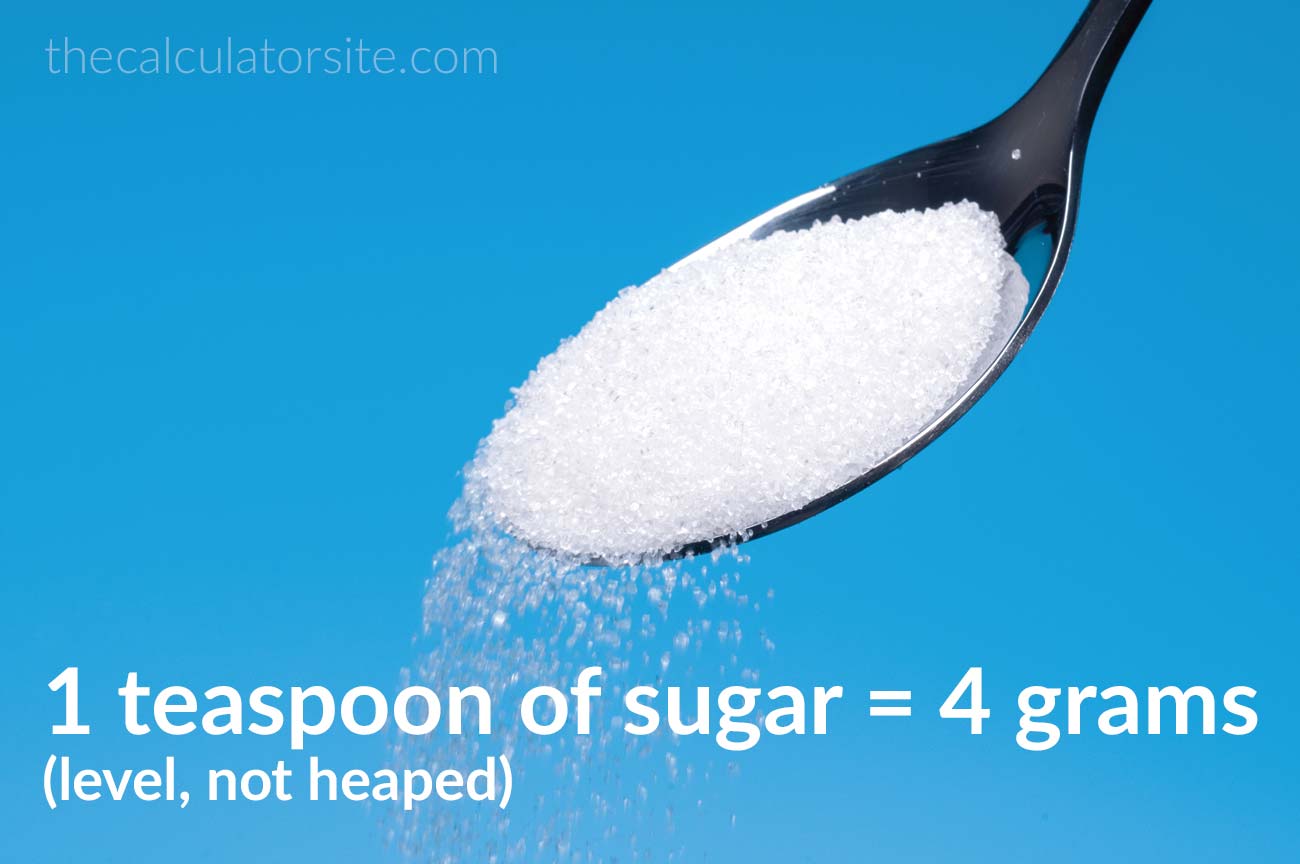


(2) Salt

| Intake | Score |
| --- | --- |
| ＜6g | 0 |
| 6-7g | 1 |
| 8-9g | 2 |
| 10-11g | 3 |
| 12-13g | 4 |
| 14-15g | 5 |
| ≥16g | 6 |
| I don't know |  |

If you choose "I don't know", please answer how do you perceive what your average taste preference is like:

- Salty
- Regular
- Un-salted


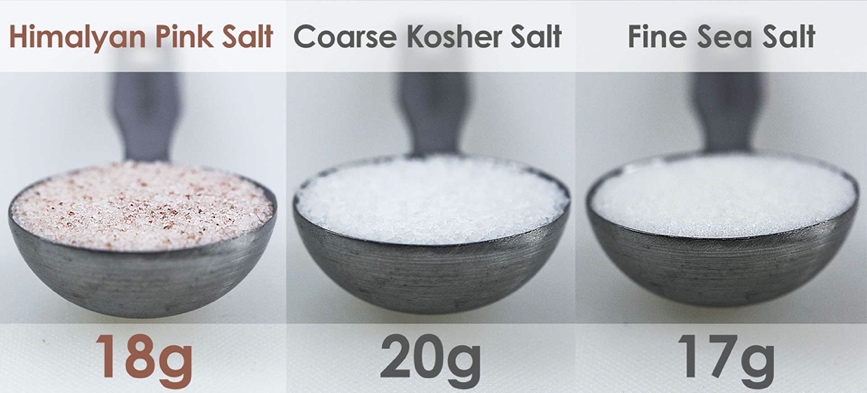


3.7. Types of food

How many kinds of food do you eat every day?

| Food types | score |
| --- | --- |
| ≥12 | 0 |
| 11 | -1 |
| 10 | -2 |
| 9 | -3 |
| 8 | -4 |
| 7 | -5 |
| 6 | -6 |
| 5 | -7 |
| 4 | -8 |
| 3 | -9 |
| 2 | -10 |
| 1 | -11 |
| 0 | -12 |
| I don’t know |  |

If you chose "I don't know", please answer how varied is the food that you eat on regular basis:

- Little variad
- Average
- Abundantly varied

3.8. Water intake (including beverages and soup)

| Intake | Score |
| --- | --- |
| ≥1200ml | 0 |
| 1100-1200ml | -1 |
| 1000-1100ml | -2 |
| 900-1000ml | -3 |
| 800-900ml | -4 |
| 700-800ml | -5 |
| 600-700ml | -6 |
| 500-600ml | -7 |
| 400-500ml | -8 |
| 300-400ml | -9 |
| 200-300ml | -10 |
| 100-200ml | -11 |
| ＜100ml | -12 |
| I don't know |  |

If you choose "I don't know", please answer what is the average amount of water that you consume per day:

- More than 6 cups (one cup 200ml, ≥6 cups)
- Around 4 and 5 cups (cup 200ml, 4-5 cups)
- Fewer than 3 cups (a cup 200ml, ≤3 cups)


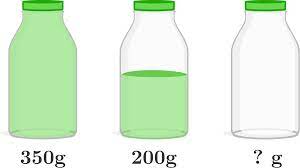


4. Sexual history **(7 factors)**

4.1. Marital status:

- Single
- Cohabitation
- Married
- Widowed
- Divorced or separated

4.2. Obstetric history:

- Number of deliveries ________
- Number of miscarriages ________

4.3. Sexual History

(1) What was the age of your sexual debut?

- 14 years old or below
- 15 - 18 years old
- 19-24 years old
- 25-29 years old
- 30 years old or older
- uncertain

(2) Number of sexual partners within the past 6 months

- None
- 1-3
- More than 3

(3) Number of sexual partners within the past 7 to 12 months

- None
- 1
- 2-3
- More than 3

(3) Total number of sexual partners in the lifetime

- None
- 1
- 2
- 3-5
- More than five

(4) How often do you use a condom when having sex?

- 0 ~ 49% of the sexual encounters
- 50% ~ 99% of the sexual encounters
- 100% of the sexual encounters

5. Substance abuse (6 factors)

5.1. Smoking amount

No ≤ 5 PCS/day >5 pieces/day

In the last month, how many days did you smoke? (Please try to estimate) __________ days

In the past 30 days, when you smoked, how many cigarettes did you smoke on average per day? (Please try to provide an estimate)

__________ / daily (one pack = 20)

5.2. Does anyone in your family/work environment have smoked for more than 6 months? (besides you)

- None
- Yes, in the room. (Select this option and continue to answer question 7)
- No, not in the house/office

5.3. How many cigarettes does the smoker in your close environment smokes while indoors per day?

__________ / daily (one pack = 20)

5.4. Drinking amount

<Once/month 1-3 times/month ≥4 times/month
