# Supplementary material for: Vaginal microbiota and personal risk factors associated with HPV status conversion—A new approach to reduce the risk of cervical cancer?
Source: PLoS One. 2022 Aug 9;17(8):e0270521. doi: 10.1371/journal.pone.0270521 (PMC9362946; doi:10.1371/journal.pone.0270521)
Supplement: S4 Text — (PDF) [file pone.0270521.s007.pdf]

The Pittsburgh Sleep Quality Index (PSQI)

Name: ____ **Date:** _______________________

Instructions: The following questions relate to your usual sleep habits during the past month only. Your answers should indicate the most accurate reply for the majority of days and nights in the past month. Please answer all questions.
During the past month,

1. When have you usually gone to bed? ___________________

2. How long (in minutes) has it taken you to fall asleep each night? ___________________

3. When have you usually gotten up in the morning? ___________________

4. How many hours of actual sleep do you get at night? (This may be different than the number of hours you spend in bed) __________________

Please check the appropriate blank below.

**5**. During the past month, how often have you had trouble sleeping because you...	**Not during the past month (0)**	**Less than once a week (1)**	**Once or twice a week (2)**	**Three or More times a week (3)**
**a**. Cannot get to sleep within 30 minutes	**a.** _______	__________	__________	__________
**b**. Wake up in the middle of the night or early morning	**b.** _______	__________	__________	__________
**c**. Have to get up to use the bathroom	**c.** _______	__________	__________	__________
**d**. Cannot breathe comfortable	**d.** _______	__________	__________	__________
**e**. Cough or snore loudly	**e.** _______	__________	__________	__________
**f**. Feel too cold	**f.** _______	__________	__________	__________
**g**. Feel too hot	**g.** _______	__________	__________	__________
**h**. Have bad dreams	**h.** _______	__________	__________	__________
**i**. Have pain	**i.** ________	__________	__________	__________
**j**. Other reason(s), please describe, including how often you have had trouble sleeping because of this reason(s):	**j.** ________	__________	__________	__________
**6**. During the past month, how often have you taken medicine (prescribed or "over the counter") to help you sleep?	**6.** ________	__________	__________	__________
**7**. During the past month, how often have you had trouble staying awake while driving, eating meals, or engaging in social activity?	**7.** ________	__________	__________	__________
**8**. During the past month, how much of a problem has it been for you to keep up enthusiasm to get things done?	**8.** ________	__________	__________	__________
	**Very good (0)**	**Fairly good (1)**	**Fairly bad (2)**	**Very bad (3)**
**9**. During the past month, how would you rate your sleep quality overall?	**9.** ________	__________	__________	__________

Physician Determined Global PSQI Score: _________
