# Supplementary material for: Vaginal microbiota and personal risk factors associated with HPV status conversion—A new approach to reduce the risk of cervical cancer?
Source: PLoS One. 2022 Aug 9;17(8):e0270521. doi: 10.1371/journal.pone.0270521 (PMC9362946; doi:10.1371/journal.pone.0270521)
Supplement: S7 Text — (DOCX) [file pone.0270521.s010.docx]

**Reproductive Tract Microecology Test Report（Metagenomics）**

Sample ID：______________ Name：_____________________ Sample Collection Date：________________

Age：_____ Phone Number：_____________________ ID Number：_______________________________

**Test Result**

Classification of the community state type (CST) microecological environment in the reproductive tract sample: ____.

CST I: In this type of reproductive tract microecological environment, bacteria from the *Lactobacillus* genus accounted for more than 50% of the microbiome composition, among which *Lactobacillus crispatus* accounted for the highest proportion. The lactic acid, acetic acid, and hydrogen peroxide secreted by Lactobacillus can effectively inhibit the growth of harmful microorganisms and create a relatively healthy micro ecological environment, which can help to reduce the risk of vaginitis and HPV infection and effectively accelerate HPV clearance.

CST II: In this type of reproductive tract microecological environment, bacteria from the *Lactobacillus* genus accounted for more than 50% of the microbiome composition, among which *Lactobacillus garneri* accounted for the highest proportion. The lactic acid, acetic acid, and hydrogen peroxide secreted by Lactobacillus can effectively inhibit the growth of harmful microorganisms and create a relatively healthy micro ecological environment, which can help to reduce the risk of vaginitis and HPV infection and effectively accelerate HPV clearance.

CST III: ﻿In this type of reproductive tract microecological environment, bacteria from the *Lactobacillus* genus accounted for more than 50% of the microbiome composition, among which *Lactobacillus iners* accounted for the highest proportion. However, the *Lactobacillus iners* have not mechanisms to protect other *Lactobacillus* genus. Hence, it’s middle status ablity to prevent cervical cancer.

CST IV: ﻿In this type of reproductive tract microecological environment, bacteria from the *Lactobacillus* genus accounted for less than 50% of the microbiome composition. The dominant harmful bacteria were *Gardnerella vaginalis*, *Atopobium vaginae* and *Dialister pneumosintes*. Since it was lack of the protection from *Lactobacillus* genus, reproductive tract microecological environment was relatively disordered to increase the risk of HPV infection, vaginitis and pelvic inflammatory. Thus, it further increased risk of preterm birth and Cervical lesions.

**Microecological Composition**

| **Microorganism Species** | **Proportion** |
| --- | --- |
| *Lactobacillus crispatus* | % |
| *Lactobacillus gasseri* | % |
| *Lactobacillus iners* | % |
| *Lactobacillus jannaschii* | % |
| *Other Latobacillus* | % |
| *Gardnerella vaginalis* | % |
| *Atopobium vaginae* | % |
| Other microorganisms | % |

**Proportion of Lactobacillus/Non-Lactobacillus microorganisms**

Lactobacillus species proportion： %

Non-Lactobacillus species proportion： %

**Harmful Microbes**

The abnormal content of other harmful microorganisms in the reproductive tract can cause sexually transmitted diseases, fungal vaginitis, bacterial vaginitis, and other gynecological diseases.

| **Harmful Microbes** | **Test Result** | **Description** | **Content** | **Reference Value** |
| --- | --- | --- | --- | --- |
| *Chlamydia trachomatis* |  | Chlamydia infection in the genitourinary tract is the most common sexually transmitted disease. The disease can transmit vertically from mother to baby through intrauterine infection, birth canal infection, and puerperal infection. Chlamydia can infect columnar epithelium, but cannot infect vaginal squamous epithelium, so it mainly causes cervical lesions and can also involve the urinary system. If left untreated, the infection will worsen, which can lead to diseases such as urethritis, cervicitis, pelvic inflammatory disease, and even worse, infertility, ectopic pregnancy, chronic pelvic pain, among other serious diseases. |  | 20.0 |
| *Neisseria gonorrhoeae* |  | Neisseria gonorrhoeae bacteria cause a purulent infection of the genitourinary system. It can also cause infections in other parts, such as the pharynx and rectum. Neisseria gonorrhoeae infection has a short incubation period and strong infectivity. Patients should receive antibacterial treatment as soon as possible. People who are susceptible to infection include those who have close contact with gonorrhea patients, those who have unprotected sex or those whose mothers have a history of gonorrhea. |  | 20.0 |
| *Ureaplasma parvum* |  | Ureaplasma parvum is a sexually transmitted microorganism, which can cause inflammation of the reproductive tract. For pregnant women, it will increase the risk of premature rupture of membranes, premature birth, chorioamnionitis, and so on. |  | 20.0 |
| *Mycoplasma hominis* |  | Mycoplasma hominis is a sexually transmitted microorganism that may cause urinary system infections and inflammation of the reproductive tract. It is associated with an increase in the pH of the reproductive tract. Long-term or repeated infections can cause infertility. Infection with this bacterium mainly manifests as inflammation of the reproductive system such as cervicitis, pelvic inflammatory disease, fallopian tube blockage, and vaginitis, and symptoms such as increased vaginal discharge and vaginal itching. |  | 20.0 |
| *Candida albicans* |  | Fungus. Generally, it does not cause disease; but in the case of excessive proliferation and decreased immunity of the patient, it can cause symptoms similar to bacterial vaginitis, that is, "vulvovaginal candidiasis", commonly known as fungal vaginitis. |  | 20.0 |
| *Gardnerella vaginalis* |  | It is one of the main pathogens that cause bacterial vaginitis, related to the increase in pH of the reproductive tract, the secretion of clue cells, and the production of ammonia-like pungent odor. This bacterium will slow down the efficiency of HPV clearance, and it may be strongly associated with an increase in cervical lesions. |  | 50.0 |
| *Prevotella bivia* |  | This microorganism is related to the increase in the pH value of the reproductive tract and the production of ammonia odor. |  | 50.0 |
| *Atopobium vaginae* |  | It is related to the elevated pH value of the reproductive tract and the generation of ammonia-like odor. |  | 50.0 |
| *Dialister pneumosintes* |  | It is related to the increase of the pH value of the genital tract, the increase of vaginal secretions, and the secretion of clue cells; it may be one of the pathogenic bacteria that cause the recurrence of bacterial vaginitis and pelvic inflammatory disease. |  | 20.0 |
| *Streptococcus agalactiae* |  | While it mainly causes asymptomatic infections, it represents a high risk for pregnant women, and its presence is associated with neonatal group B streptococcal infection. The test for Streptococcus agalactiae is an important test item during pregnancy. Newborns are very susceptible to infection as they pass through the birth canal, causing severe sepsis and meningitis. For pregnant women, in addition to causing infection, it can also cause premature rupture of membranes or late-term abortion. |  | 20.0 |
| *Timona_Prevotella* |  | It is related to increased secretions in the reproductive tract. The bacteria will also slow down the efficiency of HPV clearance. |  | 20.0 |

**Declaration**

1. This test report does not consider clinical symptoms, patient history, drug interactions, drug sensitivities, allergies, and other factors related to the patient´s medical history.
2. The results of this report are only related to the samples submitted this time, and the analysis of the results is limited to medical research and analysis of the structure of the flora. If you have any questions, please contact us within 7 working days after receiving the test results at our designated customer service line：400-605-6655.
