# Supplementary material for: Vaginal microbiota and personal risk factors associated with HPV status conversion—A new approach to reduce the risk of cervical cancer?
Source: PLoS One. 2022 Aug 9;17(8):e0270521. doi: 10.1371/journal.pone.0270521 (PMC9362946; doi:10.1371/journal.pone.0270521)
Supplement: Attachment — Submitted filename: Response reviewers comments.docx [file pone.0270521.s011.docx]

Reviewer #1: Human papillomavirus (HPV), being considered as a sexually transmitted pathogen, which is responsible for over 90% of cervical cancer cases, poses a severe threat to woman’s reproductive health. Though the factors affecting HPV-persistence are not fully understood, emerging data suggests that there exists an association between cervical HPV infections and the vaginal microbiota. This study demonstrated that several factors significantly associated with persistent HPV infection, including age, salary, history of reproductive tract infection, and the total number of sexual partners and in vaginal microbiota, Lactobacillus gasseri, Streptococcus agalactiae, and Timona prevotella bacteria may be associated with HPV clearance. Besides, by using the eHealth platform which is a user-friendly mobile application program, it is more cost-efficient than any other kind of management for researchers to pay full attention to every participant in a health-related screening program.

1. Major

Line 101 to line 104:

We found the *participant was requested to abstain from vaginal intercourse 24 hours before sampling, to wait for at least three days after menstrual blood was cleared and to avoid using vaginal douches and any vaginally administered medical treatments.* But at different stages of the menstrual cycle, such as follicular phase and the luteal phase, physiological changes could have an impact on vaginal flora growth, colonization, and community structure. It is suggested to collect sample of vaginal mucus at the same stage of the menstrual cycle or indicate what stage of the menstrual cycle the collected samples come from.

Thanks for your questions. We collected samples after 3 days beyond the menses. It can be both follicular and luteal phase because of three reasons with four references.

1.1, According to Stephanie et al, vaginal microbial diversity, as measured using the Shannon index, increased during menses blood (P < 0.001), while Lactobacillus abundances decreased (P = 0.01). Hence, we did not collect samples during menses.

1.2, Based on Bonnie et al, the overall vaginal microbiome of most women remained relatively stable throughout the menstrual cycle, with little variation in diversity and only modest fluctuations in species richness. That is to say, there is little variation between follicular and luteal phase.

1.3, The reason why it was three days accounted by Pawel et al. In their article, Figure S6 showed Shannon diversity indices over the menstrual time. Based on the figure, the diversity index decreased to one and less after three days in the end of menses.

1.4, Although samples obtained during a menstrual period should be valid, most women would prefer to obtain the sample at a time other than during their menstrual flow based on Jerome et al.

Therefore, we summarized the descriptions for: *to wait for at least three days after menstrual blood was cleared*.

2. Minor

1) Line 105 to line 106:

Sample of vaginal mucus was collected by inserting a swab into the vagina, which was then stirred/placed inside a special tube with a *preservative solution* at room temperature *until the pick-up was arranged*. Is this preservative solution a DNA protection solution?

Thanks for your questions. Exactly, this solution is a DNA protection solution through N-octylpyridinium bromide (NOPB) published in Han-Microbiome-2018 from my institution. Furthermore, the NOPB was also acknowledged by article Qian-Chin Med J (Engl)-2020

2) Line 277:

“HPV-negative to HPV-negative” would be “HPV positive to HPV negative”.

Thanks for your suggestions. It was updated in accordingly.

In summary, 6 references were added on four articles targeting your first question and two articles were targeted on the second question. They are updated both in Revised Manuscript with Track Changes file and
